# Supplementary material for: Safety Evaluation of Employing Temporal Interference Transcranial Alternating Current Stimulation in Human Studies
Source: Brain Sci. 2022 Sep 5;12(9):1194. doi: 10.3390/brainsci12091194 (PMC9496688; doi:10.3390/brainsci12091194)
Supplement: Supplementary file 1 [file brainsci-12-01194-s001.zip › brainsci-1840357-supplementary.pdf]

Supplementary Material

1. Supplemental Tables

Table S1. The statistical results of the relative change percentages of the neurological and neuropsychological measurements.

Meansurements	Active group		Sham group		Statistical results	
(Percentage)	Mean	Std. Deviation	Mean	Std. Deviation	*t*	*p*
MoCA	101.1367	6.26467	99.2815	6.14744	0.826	0.415
PPT						
Right Hand	96.0281	8.69495	92.4800	5.29532	1.494	0.144
Left Hand	94.8667	5.08215	93.9180	3.66091	0.385	0.702
Both Hands	95.4720	6.98211	94.1336	5.53225	0.788	0.436
Assembly	90.4438	6.74597	90.6322	7.74358	0.082	0.935
A-CalCAP						
SRT	102.8644	20.57278	98.0103	12.44376	0.828	0.413
CRT	97.5547	7.41878	98.4706	5.92510	-0.373	0.711
SPM1	98.4185	10.58960	101.7780	8.11183	-0.949	0.349
SPM2	110.0147	12.01190	104.3717	11.79393	1.474	0.149
NSE	89.0789	18.31330	99.0326	32.71433	-1.29	0.207
VAMS-R						
Sad	155.9458	199.38242	135.7785	169.23709	0.336	0.739
Confused	719.4068	1930.71287	549.7241	1000.58966	0.34	0.736
Afraid	684.7576	2299.92278	194.6379	351.71878	0.913	0.367
Happy	185.2106	293.18436	638.6090	2293.08975	-0.873	0.389
Tired	219.8437	382.63089	240.3417	407.93678	-0.121	0.904
Angry	143.5114	189.73218	104.8591	124.22770	0.824	0.415
Tense	809.6006	2281.36638	203.7102	224.67066	1.187	0.243
Energetic	140.3178	104.12713	173.8018	126.51480	-1.62	0.114
SAS						
Concentration	118.4211	28.81119	105.5263	18.67570	1.492	0.144

Calmness	113.8596	37.94511	107.8070	32.81567	0.526	0.602
Fatigue	90.8772	36.99503	76.8421	28.88833	1.521	0.137
Visual perception	110.0877	15.36062	106.2281	31.54383	0.265	0.793

The differences of the percentages (percentage = 100 * pre_test_value / post_test_value) between the 2 groups were tested by independent T-tests. As shown in the table, all the *p*s are larger than 0.05. These results are consistent with our statistical results that calculated with the original measure values in the Table 1. NSE: Serum neuron-specific enolase; MoCA: Montreal Cognitive Assessment; A-CalCAP: an abbreviated version of the California Computerized Assessment Package; PPT: Purdue Pegboard Test; VAMS-R: revised version of the Visual Analog Mood Scale; SAS: self-assessment scale.

Table S2. The statistical results of EEG band powers.

	Frequency bands											
**Channels**	**delta**		**theta**		**alpha**		**low beta**		**high beta**		**low gamma**	
	F	***p***	***F***	***p***	***F***	***p***	***F***	***p***	***F***	***p***	***F***	***p***
Fp1	1.452	0.233	0.890	0.450	0.194	0.900	0.119	0.949	0.580	0.629	0.230	0.875
Fp2	0.701	0.554	0.451	0.718	0.217	0.884	0.115	0.951	0.761	0.519	0.545	0.653
F7	1.094	0.356	0.370	0.775	0.078	0.972	0.065	0.978	0.186	0.906	0.285	0.836
F3	0.762	0.518	0.248	0.862	0.137	0.938	0.189	0.904	0.640	0.591	0.302	0.824
F4	0.270	0.847	0.037	0.990	0.220	0.883	0.218	0.884	0.247	0.863	0.273	0.845
F8	0.889	0.450	0.351	0.789	0.214	0.886	0.399	0.754	0.476	0.699	0.270	0.847
Fz	0.618	0.605	0.312	0.817	0.140	0.936	0.212	0.888	0.220	0.882	0.460	0.711
C4	0.243	0.866	0.486	0.693	0.320	0.811	0.318	0.812	0.301	0.825	0.710	0.549
Cz	0.838	0.476	0.761	0.519	0.234	0.872	0.368	0.776	1.508	0.218	0.327	0.806
P3	0.634	0.595	0.212	0.888	0.410	0.746	0.147	0.931	0.757	0.521	0.380	0.768
P4	0.799	0.498	1.994	0.120	0.724	0.540	1.285	0.284	1.357	0.261	1.031	0.383
Pz	0.327	0.806	0.410	0.746	1.620	0.190	0.408	0.747	1.094	0.356	0.460	0.711
O1	0.194	0.900	0.346	0.792	0.110	0.954	0.035	0.991	0.111	0.954	0.334	0.801
O2	0.243	0.866	0.486	0.693	0.320	0.811	0.318	0.812	0.301	0.825	0.710	0.549
Oz	0.594	0.621	0.391	0.760	0.225	0.879	0.143	0.934	0.391	0.760	0.309	0.819

The values shown in the table are the statistical results of 2 groups x 4 recording times rmANOVA of each channel and frequency band. All statistical significances are larger than 0.05. EEG: electroencephalograph.
